# Controversies in the management of proximal deep vein thrombosis

**DOI:** 10.5694/mja2.51796

**Published:** 2022-11-30

**Authors:** Jana‐Lee Moss, Frederikus A Klok, Uyen G Vo, Toby Richards

**Affiliations:** ^1^ Fiona Stanley Hospital Perth WA; ^2^ University of Western Australia Perth WA; ^3^ Leiden University Medical Center Leiden Netherlands

**Keywords:** Thrombosis, Thromboembolism, Embolism and thrombosis, Venous insufficiency

Future studies should focus on patient selection for interventional therapy, best practices for stent surveillance, and long term anticoagulation

Deep vein thrombosis (DVT) is a common condition affecting one in 1000 patients in the Western world.^1^ Patients normally present acutely with symptoms of a hot, painful, swollen leg. Fortunately, many DVTs remain restricted to the calf veins, with low risk of acute pulmonary embolism. Patients usually respond quickly to anticoagulation and compression, often with full recovery and no long term sequelae. However, in one‐third of patients, the DVT is located more proximal, involving the femoral and/or iliac veins.^2^ In patients with a large proximal DVT, symptoms may improve initially, but up to half of patients will report chronic symptoms.^3^ These chronic symptoms, termed post‐thrombotic syndrome (PTS) include leg swelling, difficulty wearing tight shoes or leg coverings, and difficulty walking due to aching and heaviness. In severe cases, this leads to skin changes and venous leg ulceration can occur.^2^, ^3^, ^4^ As proximal DVT can occur in relatively young and otherwise healthy people, the long term impact of PTS is significant, with reduction in quality of life^5^ and significant health care costs.^6^, ^7^

## The acute management of DVT

In patients with proximal DVT, initial management remains anticoagulation.^3^ Anticoagulation should be directed by local policy and guidelines and should be started without delay. Direct oral anticoagulants have become the standard of care worldwide in the past decade.^8^ There is no role for inferior vena cava filters in patients able to receive anticoagulation.^9^

## The role of compression stockings in proximal DVT

Compression stockings should be applied in the acute setting to promote early clot resorption, vein wall apposition, and recanalisation.^10^, ^11^ Compression stockings have traditionally been used in the long term after DVT, but the evidence of benefit is not clear. A meta‐analysis of six randomised control trials, with a weighted average follow‐up time of 38.4 months, found no benefit of compression stockings versus placebo or no stockings.^12^ This is reassuring, as patient compliance is often poor, particularly in the hot Australian climate. Two non‐inferiority studies have shown that patients without signs or symptoms of PTS after 3–6 months of compression therapy can stop wearing stockings, although those with persistent swelling may still benefit from prolonged compression therapy for up to 2 years.^13^, ^14^ We propose that compression should be individually managed. Compression can be used as a treatment to reduce symptoms of leg swelling, but there is no indication for routine long term use of compression as a preventive therapy to reduce PTS following DVT.

## The role of intervention in DVT

The past decade has seen advances in endovascular interventions for large proximal DVT, with increased availability of catheter‐directed thrombolysis (CDT), mechanical thrombolysis systems and clot aspiration/retrieval techniques to treat acute DVT, as well as intervention with dedicated venous stent systems to correct femoral, iliac, and inferior vena cava venous scarring or obstruction. These interventions have the potential to broaden existing conservative guidelines for CDT of DVT for patients presenting with limb threatening DVT (phlegmasia cerulea dolens).^8^ Thrombolysis is effective in the acute patient (with symptoms of < 14 days duration) to remove the thrombus burden, restoring the main venous flow and opening the collateral circulation (Box 1).^15^

Box 1 Thrombolysis for acute bilateral proximal deep vein thrombosis*

**Figure:**
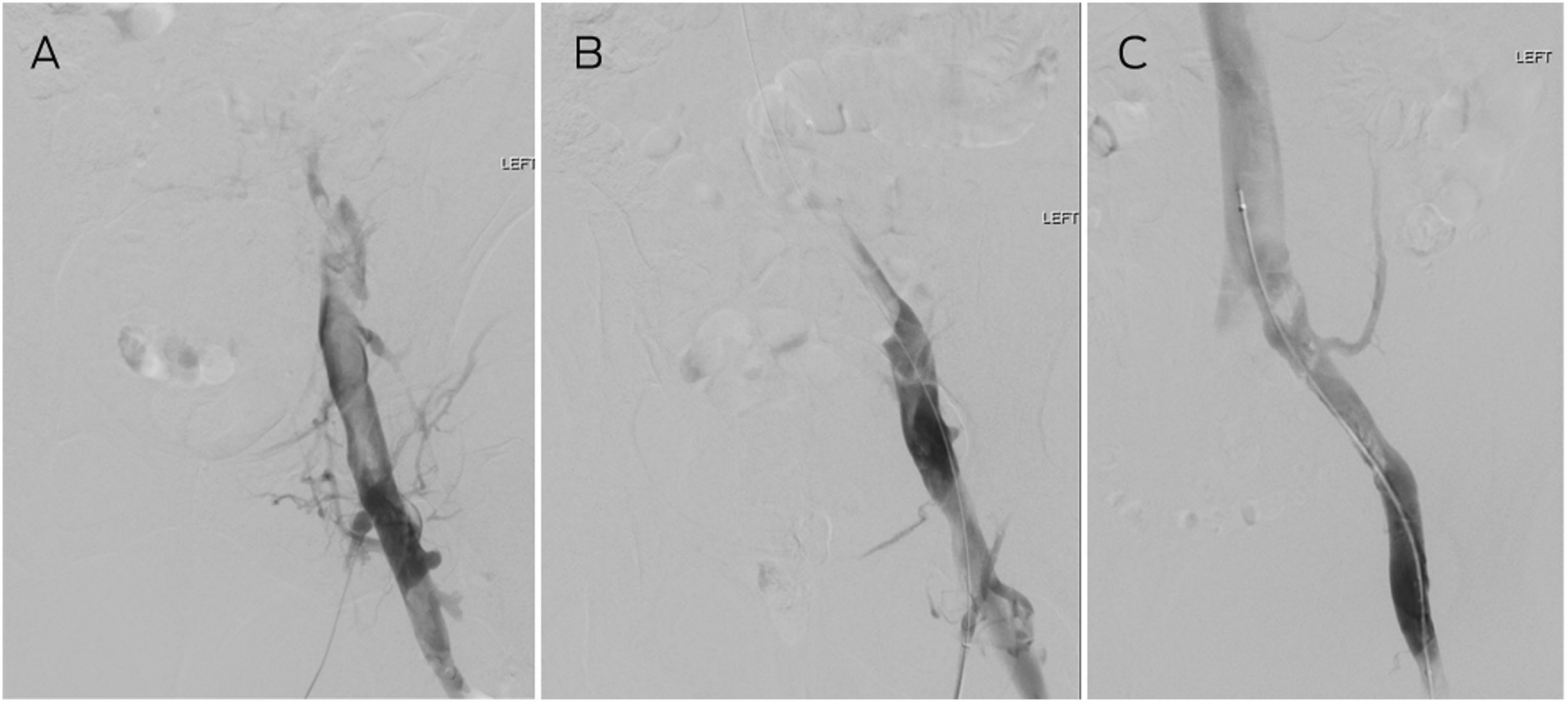
* (**A**) Venography demonstrating occlusion with filling defect in the left common femoral veins with no flow into the common iliac vein. (**B**) After 24 hours of catheter‐directed thrombolysis, the common femoral and external iliac veins have cleared, and (**C**) after 48 hours, the common iliac vein is patent with flow into the inferior vena cava. The residual filling defects (clots) resolved on anticoagulation by follow‐up scan at 6 weeks.

There have been three major randomised controlled trials on the use of thrombolysis compared with standard of care (anticoagulation and compression) in patients with proximal DVT. The CaVenT^15^ trial assessed CDT in 209 patients, from 20 centres in Norway, with symptomatic (< 21 days) DVT located higher than the proximal half of the femoral vein. The CAVA^16^ trial assessed ultrasound‐accelerated CDT in 184 patients with iliofemoral DVT at 15 centres in the Netherlands. The ATTRACT^17^ trial assessed pharmaco‐mechanical thrombolysis in 692 patients, at 56 centres in the United States, with DVT in the common femoral vein or more proximal. The primary outcome in all three trials was PTS, measured using the Villalta score, with a score > 5 denoting PTS.

Overall, CaVenT was the only trial to show benefit from interventional treatment with a reduction in the risk of any PTS (43% *v* 71%; absolute risk reduction, 28%; 95% CI, 14–42) at extended follow‐up of 5 years. Subgroup analyses of CaVenT and ATTRACT suggested benefits in patients presenting with large proximal iliofemoral DVT and in patients with severe symptoms, although the intervention did not prevent severe PTS nor improved quality of life in either trial. The general hypothesis that interventions with or without stenting would improve the long term outcome of proximal DVT was rejected by the smaller CAVA trial. Even though there was heterogeneity in the trial designs, the main outcomes suggest that intervention should not be the standard of care in all patients with proximal DVT.

Therefore, controversy exists on which patients to intervene on or whether to intervene at all in the acute setting. Our practice is to offer intervention in patients with large DVTs involving the iliofemoral veins when several days of conservative management by anticoagulation, elevation and compression has not considerably reduced the symptoms of pain or swelling. As there is a window for intervention in the first 2 weeks for thrombolysis this enables time for a trial of such conservative management and for appropriate patients to make an informed choice. Intervention should be performed in centres with experience and protocols, and which are able to offer the range of options including thrombolysis, mechanical thrombolysis or clot retrieval with internal audit and governance. A national database is in development by the Australian and New Zealand Society for Vascular Surgery, Vascular Foundation.

## The role of stenting

In patients with scarring or occlusion of the iliac veins after a proximal DVT and who have developed PTS, stenting to open the diseased venous segment can improve symptoms, although randomised control trials are lacking and evidence is scarce.^18^ In this setting, venous stenting requires a specialist staff in a centre equipped with an endovascular operating theatre and high dependency care support. The techniques of intervention differ from those of arterial intervention, as the vein can often be occluded with considerable collaterals across the pelvis (Box 2). Patient selection and informed consent is vital because reintervention rates are high and regular follow‐up is required.^19^ Patency rates are good at 80–90% at 1–2 years, although longer term (5 years) outcomes have not been widely reported. Most clinical trials, at present, represent industry licensing studies.^19^

Box 2 Venous stent placement for the management of post‐thrombotic syndrome due to occluded left iliac veins*

**Figure:**
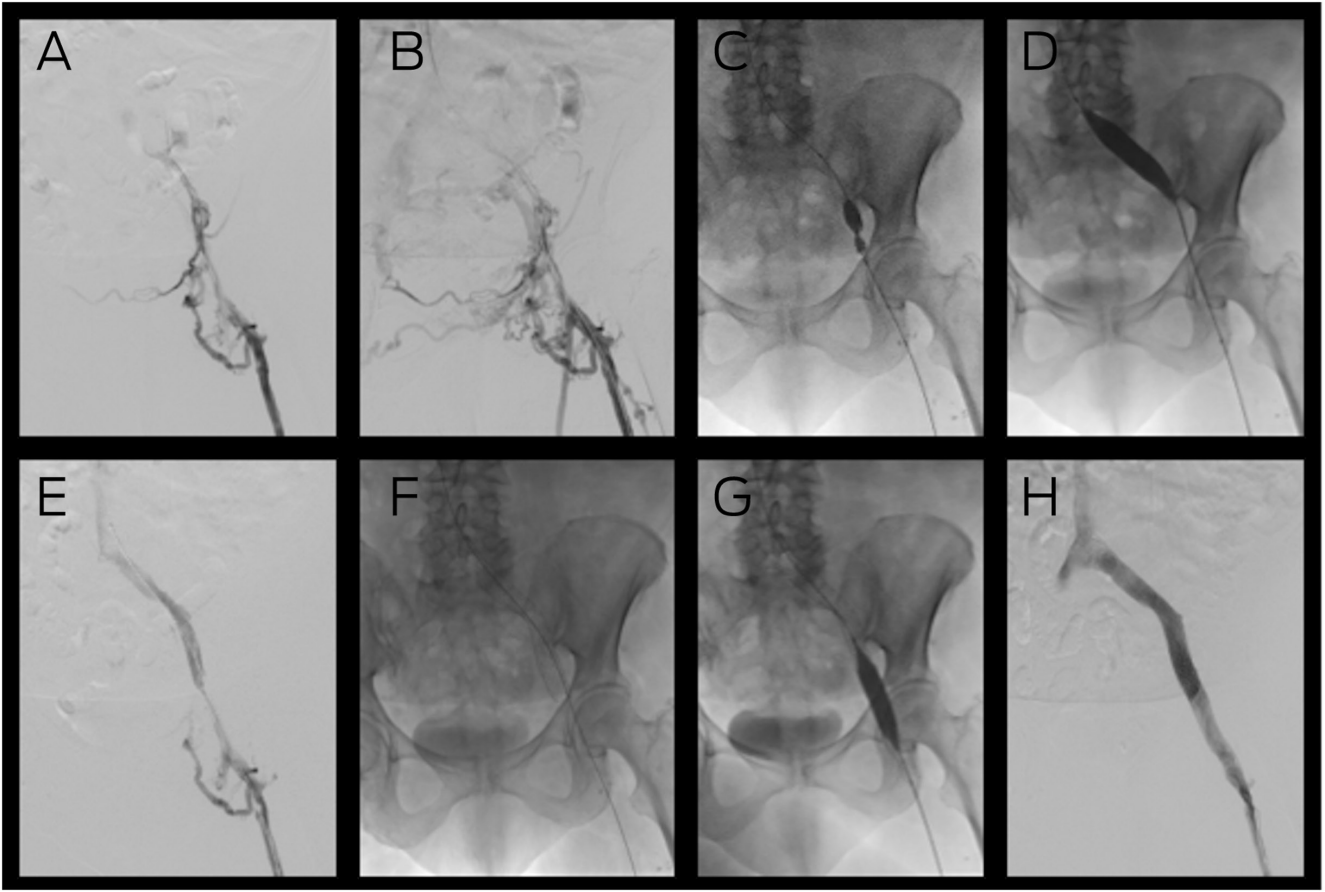
* (**A**) Venography demonstrating inflow from the femoral vein but no flow into the iliac veins, with (**B**) considerable collaterals in the pelvis. Placement of a guide wire across the lesion enabled venoplasty of the external and common iliac veins with considerable narrowing of the balloon by scar tissue (**C**) which was overcome by a high pressure (20 Atm) Kevlar 16 mm balloon (**D**). Subsequent venography (**E**) demonstrated recanalisation of the iliac vein that enabled placement of a 16 mm nitinol venous stent (**F**) from the inferior vena cava (IVC) to the common femoral vein. Following completion venoplasty (**G**), completion venography (**H**) demonstrated restoration of normal flow from the leg to the IVC.

Even though venous stenting to reopen occluded iliac veins or the inferior vena cava is being offered in an increasing number of centres, controversy exists about the role of venous stenting for patients with apparent vein compression. Compression of the left common iliac vein (CIV) under the right common iliac artery occurs as the vessels cross in the pelvis and is commonly seen as an incidental finding in 20–30% of patients lying on their back for a computed tomography scan.^20^ This may be exacerbated if the patient is dehydrated, with the venous system being underfilled and at low pressure and may just reflect a positional or functional finding. In contrast, May and Thurner^21^ described structural changes of fibrotic “spurs” in a cadaveric study in 1957. Determining between a functional compression of the CIV, that may be physiologically normal, and structural scarring of the CIV, as described by May and Thurner, is problematic. In a study of healthy volunteers who underwent venography, diagnostic signs of CIV compression and collaterals were seen in the majority.^22^ Consensus suggests that the use of intravascular ultrasound can adequately delineate vessel wall scarring.^23^ Nevertheless, there is concern that overdiagnosis of compression in patients without a history of DVT may be leading to a liberal policy of venous stenting.

The authors propose that intervention with a venous stent should be considered only with confirmed structural stenosis, scarring or occlusion confirmed by intravascular ultrasound or dynamic/augmented ultrasound in a well hydrated patient, by an experienced physician, in a dedicated referral centre. There is no current role for venous stenting in apparent compression. As with all interventions, venous stent placement does come with initial risks (bleeding or migration) and long term risk of occlusion. Therefore, the intervention should be dictated by the significance of the patients’ symptoms and compliance with antithrombotic therapy and follow‐up.

## The need for a holistic approach to DVT management

In addition to efficient diagnostic, management and therapeutic interventions, patients benefit from a holistic approach considering the whole spectrum of serious adverse events they may encounter during both the short and long term following proximal DVT. Patients should be counselled on the following aspects:

- management of bleeding risk, with identification and treatment of modifiable risk factors for bleeding;
- contraception and mitigation of anticoagulation‐associated abnormal menstrual bleeding;
- screening for antiphospholipid syndrome and underlying cancer in selected patients with unprovoked DVT;
- resuming sporting activities; and
- a general focus on maintaining a healthy lifestyle and regular exercise to prevent PTS and improve overall cardiovascular prognosis (Box 3).

Box 3 **A holistic approach to deep vein thrombosis (DVT) management**

**Figure:**
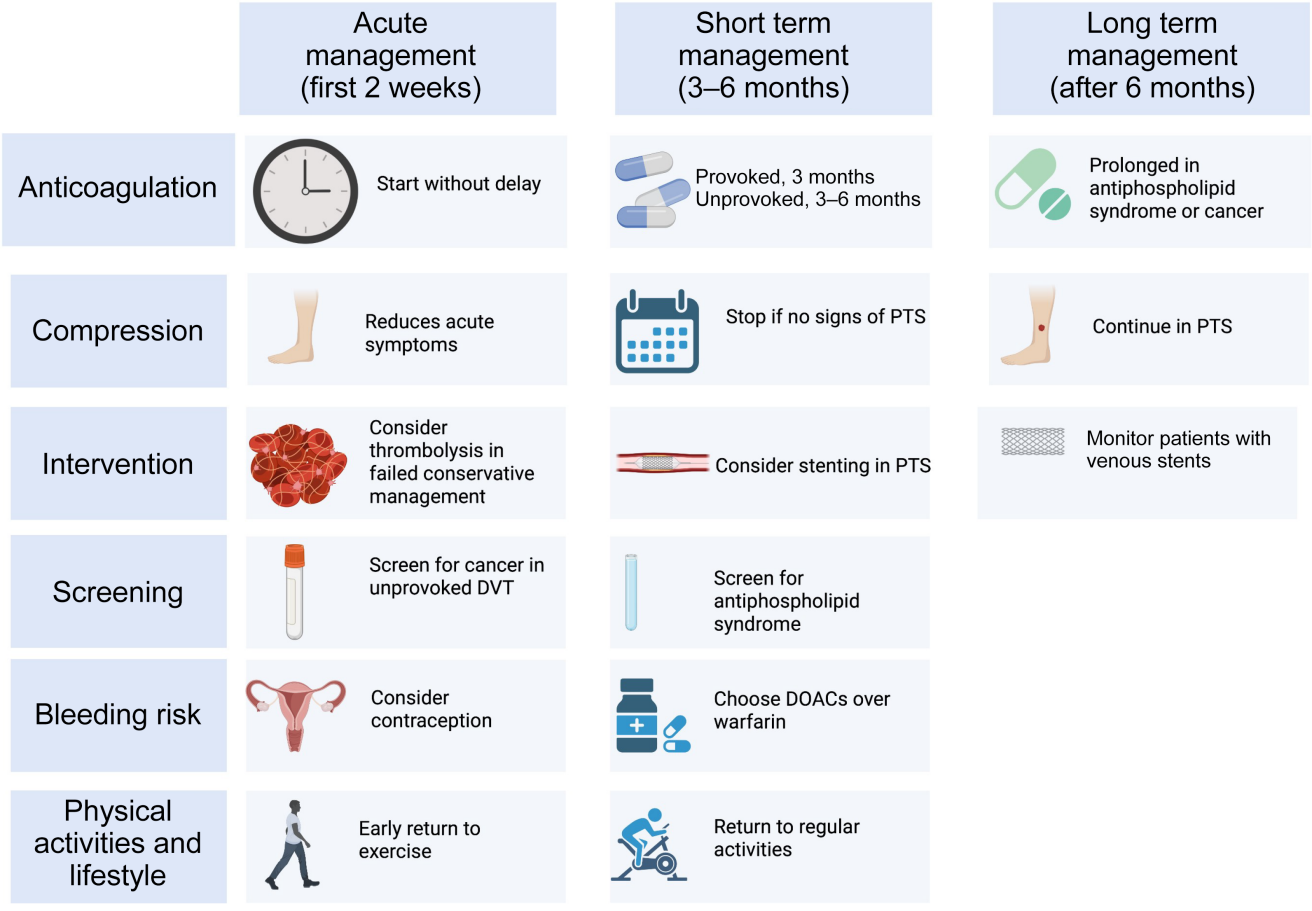
DOACs = direct oral anticoagulants; PTS = post‐thrombotic syndrome.

## Conclusion

The mainstay of treatment of an acute proximal DVT is anticoagulation, and compression stockings when they provide symptomatic relief. Selected patients may benefit from interventional treatment if early conservative management fails to resolve symptoms. In addition, patients with confirmed scarring or obstruction of the iliofemoral veins may benefit from venous stenting. Optimal patient selection for interventional therapy, best practices for stent surveillance, and long term anticoagulation after interventional procedures are key topics for ongoing and future studies. In addition to routine anticoagulation and compression therapy, we propose a holistic approach to the patient considering the whole spectrum of serious adverse events and challenges that patients with DVT may encounter.

## Open access

Open access publishing facilitated by The University of Western Australia, as part of the Wiley ‐ The University of Western Australia agreement via the Council of Australian University Librarians.

## Competing interests

No relevant disclosures.

## Provenance

Not commissioned; externally peer reviewed.
